# Oxygen Mapping within Healthy and Acutely Infarcted Brain Tissue in Humans Using the NMR Relaxation of Lipids: A Proof-Of-Concept Translational Study

**DOI:** 10.1371/journal.pone.0135248

**Published:** 2015-08-12

**Authors:** Florence Colliez, Marta M. Safronova, Julie Magat, Nicolas Joudiou, André P. Peeters, Bénédicte F. Jordan, Bernard Gallez, Thierry Duprez

**Affiliations:** 1 Biomedical Magnetic Resonance Group, Louvain Drug Research Institute, Université Catholique de Louvain (UCL), Brussels, Belgium; 2 Department of Radiology and Medical Imaging, Cliniques universitaires UCL-Saint-Luc, Brussels, Belgium; 3 Department of Neurology, Cliniques universitaires UCL-Saint-Luc, Brussels, Belgium; Fraunhofer Institute for Cell Therapy and Immunology, GERMANY

## Abstract

The clinical applicability of brain oxygenation mapping using the MOBILE (**M**apping of **O**xygen **B**y **I**maging **L**ipids relaxation **E**nhancement) magnetic resonance (MR) technique was assessed in the clinical setting of normal brain and of acute cerebral ischemia as a founding proof-of-concept translational study. Changes in the oxygenation level within healthy brain tissue can be detected by analyzing the spin-lattice proton relaxation (‘*Global T*_*1*_ *’ combining water and lipid protons*) because of the paramagnetic properties of molecular oxygen. It was hypothesized that selective measurement of the relaxation of the lipid protons (‘*Lipids T*_*1*_ *’*) would result in enhanced sensitivity of pO_2_ mapping because of higher solubility of oxygen in lipids than in water, and this was demonstrated in pre-clinical models using the MOBILE technique. In the present study, 12 healthy volunteers and eight patients with acute (48–72 hours) brain infarction were examined with the same clinical 3T MR system. Both Lipids R_1_ (R_1_ = 1/T_1_) and Global R_1_ were significantly different in the infarcted area and the contralateral unaffected brain tissue, with a higher statistical significance for Lipids R_1_ (median difference: 0.408 s^-1^; p<0.0001) than for Global R_1_ (median difference: 0.154 s^-1^; p = 0.027). Both Lipids R_1_ and Global R_1_ values in the unaffected contralateral brain tissue of stroke patients were not significantly different from the R_1_ values calculated in the brain tissue of healthy volunteers. The main limitations of the present prototypic version of the MOBILE sequence are the long acquisition time (4 min), hampering robustness of data in uncooperative patients, and a 2 mm slice thickness precluding accurate measurements in small infarcts because of partial volume averaging effects.

## Introduction

Mapping brain oxygenation is a key challenge in the clinical workup of many cerebral disorders, including acute/chronic ischemia, neoplastic processes, and a wide range of neurodegenerative disorders [1,2]. However, *in vivo* pO_2_ measurement within the brain remains an unsolved issue in clinical practice. Efficient methods for brain oxygen mapping in a clinical setting should satisfy challenging criteria such as noninvasiveness, non-toxicity (at least low radiation exposure), sufficient spatial resolution, accurate quantification of pO_2_ levels, and widespread availability, which are not by any means satisfied by currently available methods. Nowadays the reference technique for whole-brain mapping of hypoxia is Positron Emission Tomography (PET), using nitroimidazole-derived tracers which accumulate within hypoxic areas [3]. But costs, reduced availability, and radiation exposure limit its use in routine practice. Magnetic resonance (MR) techniques using endogenous contrast agents (CA) could be alternatives to PET for oxygen levels measurements [4–6]. Endogenous sources of O_2_-driven tissue contrast in MR Imaging include variations in T_2_* (effective transversal relaxation time) and T_1_ (spin-lattice or longitudinal relaxation time) [7]. T_2_* is sensitive to the deoxyhemoglobin (Hb)/oxyhemoglobin (HbO_2_) ratio within vessels [8]. T_2_* mapping, also referred to as ‘*functional MR imaging*’ or ‘*blood oxygen level dependent* (BOLD) *MR imaging* ‘is mainly sensitive to variations in intravascular oxygenation and has been successfully applied to monitor changes in the oxygen level within the vascular compartment, e.g. in neuronal activation. But BOLD-MRI has demonstrated significant limitations in terms of quantitative relationships between response signal intensity and true pO2 changes within tissues, and in terms of sensitivity to changes in total hemoglobin content [9]. In addition, the BOLD-based approach to brain oxygenation measurement has specifically demonstrated additional limitations such as excessive dependence on B_0_ inhomogeneities, water diffusion, and ultra-structural properties of the capillary network [10]. T_1_ is sensitive to dissolved molecular oxygen within tissues, thereby acting more as a tissular T_1_- shortening paramagnetic CA than an intravascular one [11]. Analysis of proton T_1_ relaxation induced by molecular oxygen has shown that it can be used to monitor changes in tissue oxygenation in solid tumors and several normal tissues such as the spleen, liver, renal cortex, and skeletal muscles [12,13]. The technique referred to as ‘*Oxygen-enhanced MRI’* measures the proton T_1_ relaxation time, which is mainly influenced by the water protons. We called this measurement ‘Global T_1_’ in the present study. Recently, the MOBILE (Mapping of Oxygen by Imaging Lipids relaxation Enhancement) sequence was developed to increase the sensitivity of T_1_ measurements by selectively recording the T_1_ of the lipids, a specific measurement we called ‘Lipids T_1_‘. This method is conceptually based on the higher solubility of oxygen in lipids than in water and the demonstrated ability to monitor changes in tissue oxygenation in brain, muscles, liver and tumors in a preclinical setting [14]. Moreover, a significant correlation has been established between Lipids R_1_ (with R_1_ = 1/ T_1_) values (obtained with MOBILE) and pO2 values recorded with Electron Paramagnetic Resonance [15], suggesting the quantitative value of MOBILE in this pre-clinical setting. In a translational ‘proof-of-concept’ study, we have attempted to demonstrate the applicability of MOBILE in healthy volunteers and in acute stroke patients using standard clinical MR equipment. In the second cohort we investigated whether it is capable of detecting brain oxygen deprivation in the paradigmatic pathological condition of acute decrease in oxygen supply within a lipid-rich biological tissue.

## Materials and Methods

### Healthy volunteer and patient cohorts

After the study protocol was cleared by the institutional Ethics Committee (Commission d’Ethique Biomédicale Hospitalo-Facultaire, Medical Faculty of Université Catholique de Louvain, Brussels, Ref CE 2011/21JUIL/255Belgium no. 403201111606) an initial normative cohort of 12 healthy volunteers was examined after giving their written informed consent. A standardized MR imaging protocol including a FLAIR sequence together with Global R_1_ (= 1/T_1_), Lipids R_1_, and R_2_* (= 1/T_2_*) sequences was applied to all of them using the same 3T clinical MR system. Eighteen patients presenting with acute ischemic stroke were then recruited. All potential candidates for inclusion were duly informed that the study protocol was investigational, without impact on either individual treatment strategy or prognosis, and that no parenteral contrast agent (CA) would be administered. All of them gave their written consent. Commitment was paid to avoid any interference with the individual therapeutic strategy.

Criteria for inclusion were: i: the presence of a large infarction on the admission brain CT or MR workup; ii: feasibility of the study MR examination within a 2- to 3-day delay after stroke onset; iii: absence of prior thrombolytic therapy, to minimize risk of confounding micro-hemorrhages; and iv: informed consent for the study protocol obtained from patients and/or a decision-maker substitute.

Criteria for exclusion were: i: bleeding, mainly secondary hemorrhagic transformation of initially ischemic stroke; ii: presumptive poor cooperation and/or confusion/agitation/incoercible motion.

### MR imaging

All examinations were performed on the same clinical 3-Tesla MR system (Achieva 3T Release 3.2, Philips Healthcare, Best, The Netherlands) using a 32-channel receiver head coil. A standardized protocol which included whole-brain covering fluid-attenuated inversion-recovery (FLAIR), Echo-Planar Imaging gradient echo T_2_ (EPI-T_2_*), and diffusion-weighted imaging (DWI) sequences together with the three investigational Global R_1_, Lipids R_1_ (MOBILE), and R_2_* acquisitions was applied to all volunteers and patients. Since all three of the latter sequences allowed data acquisition at only one slice location, the ‘best’ slice location was defined as that displaying the largest infarcted zone without hemorrhagic transformation on morphologic FLAIR/DWI/EPI-T_2_* images. Parameters of conventional sequences were as follows: (i) FSE-FLAIR: TR: 10,000 ms; Ti: 2,800 ms; TE:125 ms; Sense factor: 1.5; slice thickness: 4 mm; (ii) DWI: TR: 2907 ms; TE: 55 ms; Sense factor: 2; b factors: 0–1000 mm^2^/s; slice thickness: 4 mm; (iii) EPI-T_2_* sequence: TR: 1461 ms; TE: 16.11 ms; EPI factor: 9; Flip angle: 40°; slice thickness: 4 mm.

Parameters of *investigational* sequences were as follows:

- for Global R_1_ measurements: a ‘Look Locker’ T_1_ TFE sequence with TR/TE/flip angle/TFE/NSA = 3.467 ms/1.45 ms/5°/10/1 was applied for an acquisition time (AT) of 10s to obtain one 20mm-thick slice covering a FOV of 183x230 mm with a matrix size of 80^2^ resulting in a voxel size of 3.91x5.08x20 mm.
- for Lipids R_1_ measurements (MOBILE): a similar sequence was used with the addition of a 90° SPIR (SPectral saturation by Inversion Recovery) pre-pulse to spoil water with a bandwidth of 300Hz centered on the water peak; 38 images averaged 30 times with metrics similar to those for the previous acquisition were obtained for an AT of 4 min.
- for R_2_* measurements: a multi Fast Field Echo (mFFE) sequence was performed with TR/flip angle/echoes = 250ms/18°/15 to acquire 32 4mm-slices for a total acquisition time of 41.8 s. The FOV was 230X183 and the matrix size 320^2^, resulting in a voxel size of 0.9X1.12X4 mm.

### Data analysis

Data were processed using self-programmed Matlab scripts (MatLab Release 2013a, The MathWorks Inc., Natick, MA, USA). Parametric maps were reconstructed for Global R_1_, Lipids R_1_, and R_2_* data. Two-level filtering was applied for T_1_ measurements: i: T_1_ values were deleted when the relative error made on this value exceeded 30%; and ii: T_1_ values below 100 ms were considered not characteristic for lipid protons and therefore were also deleted [16]. For each patient, the infarcted area was delineated on FLAIR images by manual planimetric contouring around the hyperintense region and a contralateral mirror- ROI within unaffected brain tissue was manually generated. To be consistent with previous reports dealing with oxygen-enhanced MRI [12–15], the results were given in terms of R_1_ values instead of T_1_. Both ROIs were electronically superimposed on parametric R_1_ and R_2_*maps. Median values of relaxation rates within the two ROIs (infarct versus mirror-ROI) were calculated. In volunteers, each cerebral hemisphere was analyzed as a whole using the same routine.

### Statistical analysis

Analysis of differences between ischemic brain tissue and unaffected contralateral brain tissue was made for each parameter using the GraphPad Prism Software (GraphPad Software Inc., Release 5, San Diego, CAL, USA). Normality of datasets was assessed using the D’Agostino & Pearson test with p<0.05 rejecting the normality. The parametric paired *t-*test in cases of normal distribution of the R_1_ and R_2_* values and the non-parametric Wilcoxon test for matched pairs in cases of non-normal distribution were performed to compare medians obtained from ischemic and non-ischemic areas. Similarly parametric unpaired *t*-tests and non-parametric Mann-Whitney tests were performed to compare median values within unaffected mirror ROIs in stroke patients versus normal brain in healthy volunteers.

## Results

### Study design: volunteer and patient recruitment and data analysis

Twelve volunteers were recruited to obtain a normative database of basal Global R_1_, Lipids R_1_, (MOBILE) and R_2_* values in healthy brain tissue. Thereafter, 18 consecutive patients presenting with acute (48–72 hours after onset of symptoms) brain ischemic stroke were recruited. Both groups were examined after giving informed consent according to the institutional Ethics Committee (EC) requirements. Seven patients had to be removed from the database because of uncontrolled motion, one for improper infarct analysis because of a small infarction of which analysis was degraded by partial volume averaging with cerebral spinal fluid (CSF), and two patients with lacunar infarcts including less than 10 voxels from which accurate R1 measurement was calculable. As a result, only 8 patients were finally included in the study. Among them, one patient had two concomitant acute infarcts resulting in 9 ischemic areas available for analysis, 8 of which were located in the supratentorial space and one in the posterior fossa. None of the patients had received thrombolytic therapy or had hemorrhagic transformation of infarcted brain tissue at the time of the MR examination.

### Typical maps & analysis

**Fig 1:**
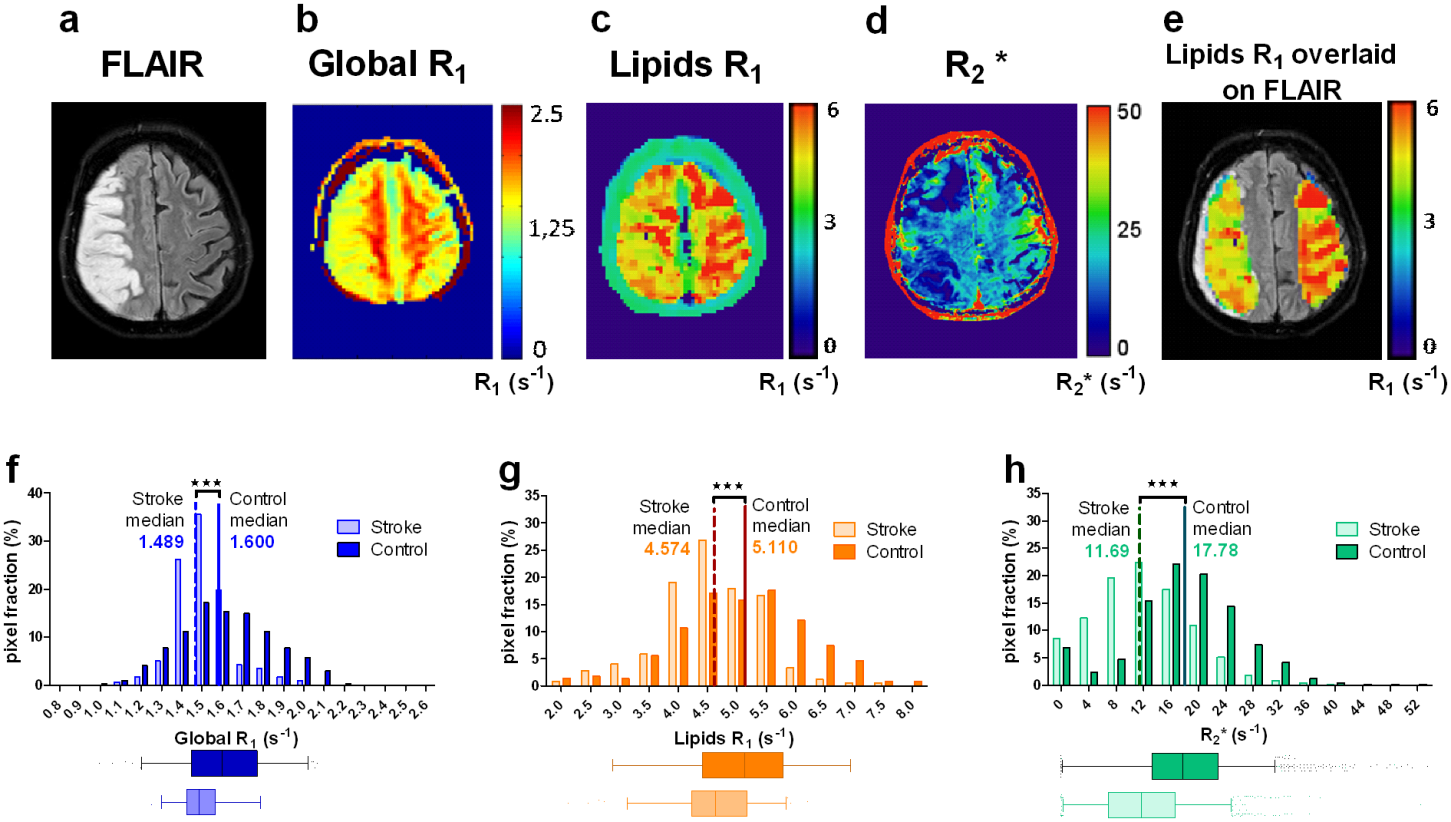
Full display of morphological/quantitative data sets obtained in a patient. Axial transverse FLAIR image (**1.a**) through centrum semiovale showing hyperintensity within acutely infarcted whole territory of the right middle cerebral artery (MCA), and corresponding global R_1_-mapped image (**1.b**), lipids R_1_-mapped image (MOBILE) (**1.c**) and R_2_*-mapped image (**1.d**) in a similar slice location. Image 1.e: overlay of the two ROIs (right-sided contour of infarction and left-sided mirrored one) from the MOBILE map (**1.b**) on the FLAIR image (**1.a**). Images 1b-1e have been smoothed using the ‘Gaussian Blur’ function (radius of 2 pixels) of the GIMP software (GIMP 2, GNU Image Manipulation Program, GPLv3). Histograms of global R_1_ (**1.f**), lipids R_1_ (**1.g**) and R_2_* (**1.h**) of the same patient demonstrating significant shift of the medians to lower values for the stroke area. Differences of 0.111 s^-1^, 0.536 s^-1^ and 6.09 s^-1^ are observed for global R_1_, lipids R_1_ and R_2_*, respectively.

Fig 1 displays a morphological axial FLAIR image (Fig 1b) together with parametric maps for Global R_1_ (Fig 1b), Lipids R_1_ (MOBILE) (Fig 1c), and R_2_*(Fig 1d) in a similar slice location. Analysis was performed on manually delineated regions of interest (ROIs) contouring the infarcted area and the mirrored area within the unaffected contralateral hemisphere on the morphological image, as illustrated by the overlay of the two ROIs from the MOBILE R_1_ map of the FLAIR image (Fig 1e). Individual histogram analysis (Fig 1f–1h) demonstrated a significant shift to lower values of the medians within infarcted tissue when compared to contralateral unaffected brain tissue for Global R_1_ values (Fig 1f), Lipids R_1_ values (Fig 1g), and R_2_* values (Fig 1h). The statistical results for each patient are presented in Table 1.

**Table 1:** Statistical details: statistical tests were performed depending on results of the D’Agostino & Pearson test for the normality of the data distribution with a parametric test (classic) used in cases of normal distribution, and non-parametric tests (bold and italic) used in cases of non-normal distribution. Calculations were performed using paired tests when both datasets were obtained from the same patient. The p values are given for each patient (P#X).

**1. Data from stroke vs mirrored normoxic area in each patient**. Paired test: Non-parametric (Wilcoxon matched paired test), or ***Parametric test (Paired t-test)***		
**Global R1**	**Lipids R1**	R2*
P#3: p<0.0001	P#3: p <0.0001	P#3: p <0.0001
P#6: p<0.0001	P#6: p = 0.1148	P#6: p<0.0001
P#6b: p<0.0001	P#6b: p = 0.0091	P#6b: p<0.0001
P#9: p<0.0001	P#9: p = 0.0550	P#9: p<0.0001
P#11: p<0.0001	***P#11*: *p = 0*.*1459***	P#11: p<0.0001
P#12: p = 0.0183	P#12: p = 0.0261	P#12: p = 0.0797
***P#13*: *p<0*.*0001***	***P#13*: *p = 0*.*2457***	P#13: p<0.0001
***P#15*: *p<0*.*0001***	P#15: p = 0.0347	P#15: p = 0.0449
P#17: p = 0.9224	***P#17*: *p = 0*.*1556***	P#17: p<0.0001
**2. Medians in stroke vs mirrored normoxic areas**. Paired test: Non-parametric (Wilcoxon matched paired test), or ***Parametric test (Paired t-test)***		
**Global R1**	**Lipids R1**	R2*
***p = 0*.*027***	***p<0*.*0001***	p = 0.0195
3. **Medians in patient’s normoxic areas vs volunteers’ healthy brain tissue**. Unpaired test: Non-parametric test (Unpaired t-test), or ***Parametric test (Mann-Whitney test)***		
**Global R1**	**Lipids R1**	R2*
***p = 0*.*2511***	***p = 10*.*99***	p = 0.1757

### Pooled data analysis of the whole patient group

**Fig 2:**
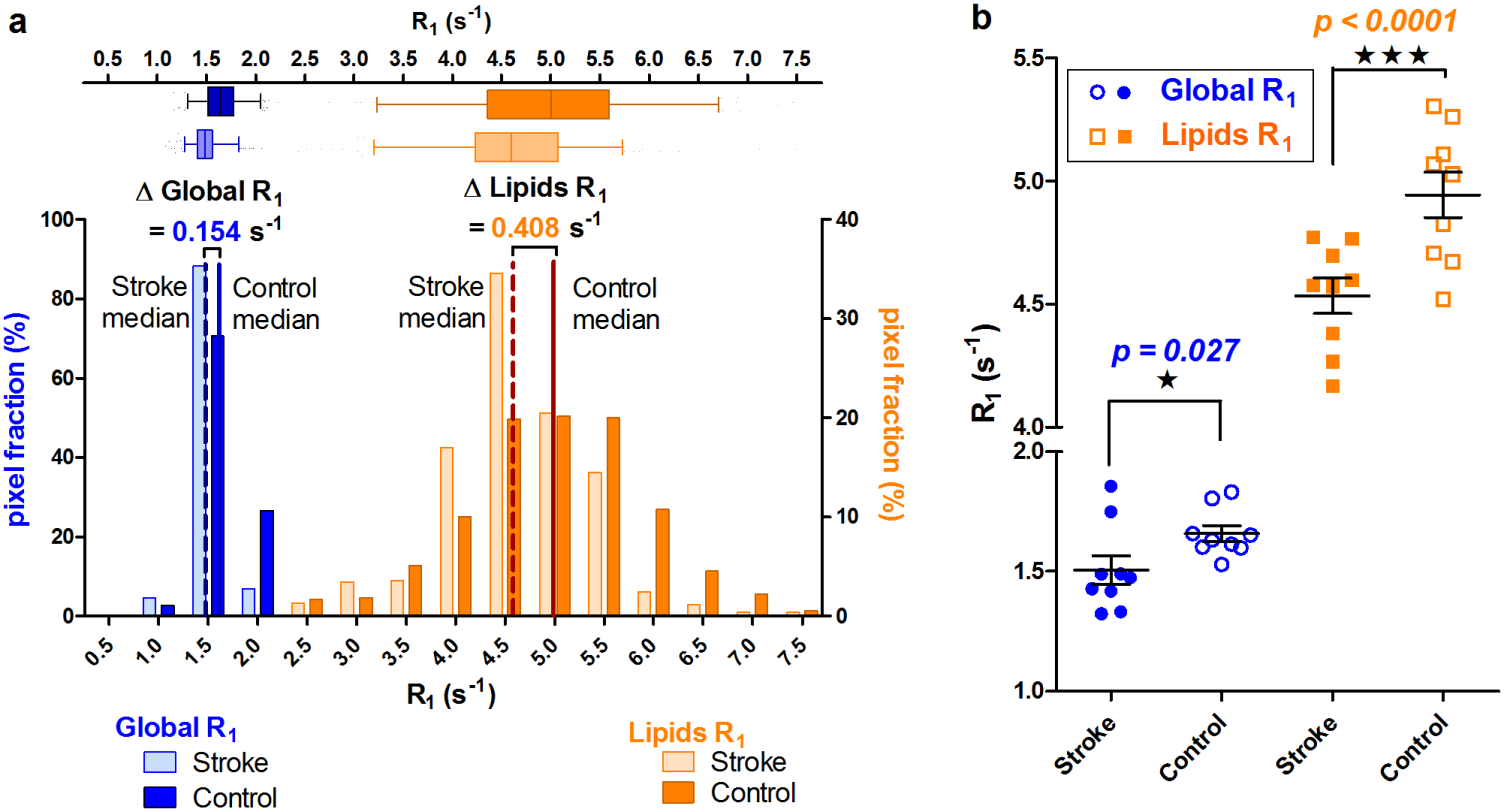
Comparison between infarcted and mirrored normoxic areas in patients. Histogram and scatter-plot of pooled 9 infarcted areas (‘stroke’) versus unaffected mirror areas (‘control’). **3.a**: Histograms demonstrated that the shift to lower values of lipids R_1_ in stroke areas (0.408 s^-1^; p<0.0001) is more pronounced than the corresponding shift observed with global R_1_ values (0.154 s^-1^; p<0.027). **3.b**: Scatter plots demonstrated a greater difference between medians for lipids R_1_ at 0.409 s^-1^ with p <0.0001 (in orange) than for global R_1_ at 0.151 s^-1^ with p<0.027 (in blue).

Pooled data histogram analysis of the 9 infarcts (Fig 2) confirmed the significant shift of the medians within ischemic tissue to lower values for Global R_1_and for Lipids R_1_ when compared to unaffected brain tissue. A 0.154 s^-1^ mean difference was calculated between medians for Global R_1_ within infarct ROIs and contralateral mirrored ROIs, resulting in a p value = 0.027. A 0.408 s^-1^ difference was calculated for Lipids R_1_, resulting in an improved p value < 0.0001 (Fig 2b). A statistically significant shift to lower values was also observed for the medians of the R_2_* values upon group analysis, with a p value of 0.0195, in an overall range similar to that for individual analyses.

### Comparison between stroke patient group and normative healthy volunteer cohort

**Fig 3:**
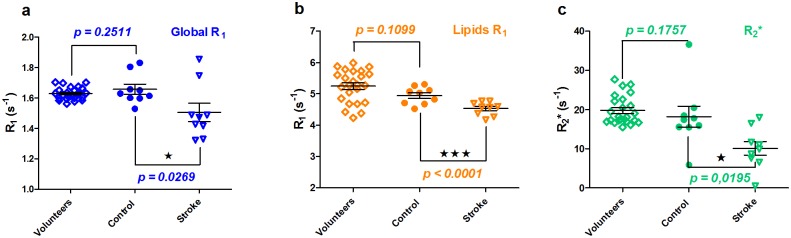
Healthy volunteers’ brain tissue (‘Volunteers’) versus unaffected mirror-areas in stroke patients (‘Control’) versus infarcts (‘Stroke’). Global R_1_ plots (3.a), Lipids R_1_ plots (3.b) and R_2_* plots (3.c) demonstrated a statistically significant difference between infarcts and unaffected mirror-ROIs in patients. In turn, no difference was observed for all modalities between the values obtained in patient mirror-ROIs and healthy brain tissue in volunteers.

Fig 3 shows a close correlation between medians of contralateral mirror ROIs within stroke patients’ unaffected brain tissue (‘control’) and healthy brain hemispheres of volunteers (‘volunteers’) for Global R_1_, Lipids R_1_, and R_2_* values (p > 0.10).

## Discussion

*In vivo* pO_2_ measurement within tissues is a crucial and unsolved challenge in clinical practice. Standard oximetry methods involve either traumatic (deeply implanted electrodes) or irradiating (PET) techniques, routine use of which therefore seem precluded. This study investigated whether a new non-invasive and innocuous technique could highlight hypoxia in the paradigmatic condition of acute ischemic stroke. The MOBILE sequence using molecular oxygen as an endogenous CA was designed for noninvasive measurements of tissue R_1_ [14]. The ability of the technique to monitor an increase in brain oxygenation during a hyperoxic challenge [14] as well as positive and negative changes in oxygenation in preclinical tumor models has been previously demonstrated [15]. This preliminary translational implementation was attempted in the brain because of the exquisite dependence of brain tissue viability on O_2_ supply and because of the elevated lipid content of cerebral tissue. Moreover, stroke is a paradigmatic condition in which both normoxic and hypoxic areas are displayed on the same axial transverse slice of the brain.

The results confirmed the ability of MOBILE to highlight hypoxia in a stroke area when compared to a normoxic brain area. R_1_ values recorded in ischemic areas were significantly lower than those calculated within contralateral unaffected areas for both Global R_1_ (which was consistent with existing literature [17,18]) and Lipids R_1_. Although both methods seemed able to distinguish hypoxic from normoxic areas, a slightly more significant performance was observed for selective lipid enhancement. The true oxygenation status within injured brain tissue could not be assessed quantitatively by measuring differences between Lipids R_1_ values within stroke vs. normoxic mirrored areas, thereby precluding diagnostic accuracy and/or prediction of patient outcome. Indeed, the difference between Lipids R_1_ values measured in stroke areas and Lipids R_1_ values measured in contralateral normoxic brain was not sufficient to predict the oxygenation status from a Lipids R_1_ measurement: in Fig 2, the lowest values of Lipids R_1_ obtained in the ‘control’ areas in fact overlapped with the highest values measured in ‘stroke’ areas. This was also observed with conventional R_1_ measurements. Several studies have shown that Global T_1_ measurements are strongly influenced by water content [19,20]. Consequently the Global T_1_ values calculated within the stroke area result from the opposing influences of edema vs. hypoxia. Since the MOBILE sequence included a saturation pulse spoiling the water signal, the Lipids T_1_ should be less influenced by the change in water content within the infarcted area, even if we cannot neglect this parameter. In addition to the water content, it has been demonstrated that blood flow [12,21], basal oxygenation and basal saturation of hemoglobin [10] are other factors influencing the relaxation rate measurement. From our results, we cannot exclude that those parameters may have influenced the T_1_ measurements. Nevertheless, preclinical studies have shown that the MOBILE technique is sensitive to positive and negative changes in oxygenation, and the quantitative capabilities of the technique have been demonstrated in a preclinical setting [15]. Consequently, despite the multiple factors influencing Lipids R_1_ values, the difference between Lipids R_1_ values measured in hypoxic areas (infarcted brain) and Lipids R_1_ values measured in contralateral unaffected brain were considered to mainly reflect a difference in tissue oxygenation. To assess the reliability of Lipids R_1_ measurements, we compared R_1_ values within contralateral unaffected brain tissue of stroke patients to those recorded in healthy brain tissue of volunteers and failed to demonstrate significant differences between the two groups. This correspondence suggested the consistency of MOBILE-based brain pO_2_ mapping in distinguishing hypoxic from normoxic areas displayed in the same slice location in the axial-transverse plane. However, further work needs to be done to test the repeatability and to investigate the quantitative aspect of MOBILE in a clinical setting. Each patient in the study was his/her own control since the symmetrical anatomy of the brain had the advantage of allowing accurate comparison between ischemic tissue and unaffected tissue in a similar area within the contralateral hemisphere.

The present version of the MOBILE sequence suffered from practical limitations at acquisition. In particular, a 4-minute acquisition time precluded its use in uncooperative and/or neurologically disabled patients, and a 20-mm slice thickness resulted in partial volume averaging artifacts which were deleterious e.g. in the upper brain areas where subcutaneous fat superseded the signal from brain lipids. Similarly, MOBILE did not allow accurate measurements in juxtaventricular regions because of partial averaging effects. In addition, significant dispersion of Lipids R_1_ measurements was observed in the processing phase within the unaffected contralateral mirror ROI in patients or within healthy brain tissue in volunteers. Finally, fitting was not possible in some voxels, resulting in a loss of information (6% to 37% of voxels were excluded from the MOBILE analysis after the application of the two filters), which constituted a synergistic penalty with partial averaging for small infarct analysis.

A reduction in the median values of R_2_* was also observed when using BOLD-MRI to compare ischemic and non-ischemic tissue. This feature may sound paradoxical, since many reports have highlighted a shift to higher values of R_2_* values in hypoxic areas [22,23]. However, a few hours after initiation of the stroke process, the briefly delayed onset of vasogenic edema increases T_2_* values independently of blood oxygenation effects, resulting in a decrease in R_2_*. Moreover, electron-nuclear interactions between neighboring atoms give rise to a dissipative relaxation mechanism described by T_2_ which is closely linked to T_2_* [1]. Thus far, it has been expected that a shift to lower values of R_2_* would be recorded within infarcts in a cohort of patients who were examined between 48 to 72 hours after stroke onset. This difference might appear to be more accurate than differences recorded with Lipids R_1_ or Global R_1_measurements. But R_1_ and R_2_* measurements do not reflect similar pathophysiological sub-processes, since R_1_ globally assesses *tissue* oxygenation and R_2_* mainly assesses *intravascular* imbalance of the ratio between oxyhemoglobin and deoxyhemoglobin, which indirectly reflects insufficient O_2_ supply. R_1_ and R_2_* are therefore hypothesized to provide complementary information.

Several quantitative magnetic resonance techniques are available to measure tissue oxygenation. Electron Paramagnetic Resonance (EPR) allows real-time monitoring of oxygenation, but it is an invasive method requiring the injection of a paramagnetic probe [24]. The relaxation of the paramagnetic probe quantitatively reflects the partial pressure of oxygen. However, EPR is not widely available, has limited depth of efficiency, and only allows spectroscopic measurement, without image acquisition. 19F-MRI is a noninvasive method able to follow positive and negative changes in oxygenation after the administration of an emulsion of perfluorocarbon [25,26]. However, to our knowledge, there is no study reporting the clinical application of 19F-MRI since the perfluorocarbons are still awaiting FDA approval. The mqBOLD method should probably be the most relevant quantitative technique available for clinical implementation. This method allows quantitative mapping of tissue oxygenation by combining information provided by standard sequences aimed at measuring the blood volume fraction, the field inhomogeneities and the tissue T_2_ before and after the administration of a contrast agent [27]. mqBOLD has already been successfully applied in gliomas and stroke models [28]. Tissue pO_2_ estimates obtained in a non-traumatic and innocuous way using the MOBILE sequence running on routine clinical equipment could add to the brain perfusion information which reflects the tissue vascularization more than the true oxygenation level. The exploratory implementation of MOBILE reported here in a clinical setting was not designed to provide diagnostic accuracy in cases of hyperacute stroke but to assess whether MOBILE could demonstrate hypoxia within infarcted brain areas as a proof- of-concept in humans, as it is ethically unfeasible to make direct measurements in normal brain tissue. With the added support of pre-clinical published results, we may then extrapolate the use of MOBILE in tumor conditions. The application of MOBILE in patients with brain tumors would be of great interest in planning radiotherapy, since tumor hypoxia induces radioresistance, and since killing hypoxic tumor tissues requires the administration of a radiation dose 3 times higher than that in normoxic tumor tissue. The MOBILE technique could be therefore a sensitive and noninvasive method for radiotherapy planning, delineating the tumor hypoxic regions that should benefit from a boost of irradiation. Further studies will investigate this application.

The initial steps of clinical applicability and consistency of results have been successfully completed. Further refinements of the sequence yielding higher spatial resolution and enhanced accuracy of quantitative results are being implemented.

## Conclusion

In conclusion, feasibility of MR-based *in vivo* brain pO_2_ mapping through calculation of the relaxation effect of molecular O_2_ on water and lipid protons was demonstrated in both normal cerebral tissue and acutely infarcted tissue using a clinical 3-Tesla equipment. The MOBILE technique selectively sampling relaxation effects of O_2_ on lipid protons was able to pinpoint hypoxia in infarcted areas compared to normoxic brain tissue. The availability of such an innocuous and repeatable technique for tissue pO_2_ mapping on routine MR systems opens up extensive prospects for investigation in clinical practice.
